# Protective effect of Cl-amidine against CLP-induced lethal septic shock in mice

**DOI:** 10.1038/srep36696

**Published:** 2016-11-07

**Authors:** Ting Zhao, Baihong Pan, Hasan B. Alam, Baoling Liu, Roderick T. Bronson, Qiufang Deng, Erxi Wu, Yongqing Li

**Affiliations:** 1 Department of Surgery, University of Michigan Hospital, Ann Arbor, MI, 48019, USA; 2 Rodent Histopathology Core - DF/HCC - Dana-Farber Cancer Institute, Harvard Medical School, Boston, MA, 02115, USA; 3 Department of Neurosurgery, Baylor Scott and White Health, Temple, Texas, 76508, USA

## Abstract

Production of innate and adaptive immune cells from hematopoietic stem cells, and maturation of T lymphocytes are effective immune responses to fight severe microbial infection. In sepsis, this emergency myelopoiesis is damaged, leading to failure of bacterial clearance, and excessive stress-induced steroids cause immature T-lymphocyte apoptosis in thymus. We recently found that Cl-amidine, a peptidylarginine deiminase (PAD) inhibitor, improves survival in a mouse model of cecal ligation and puncture (CLP)-induced septic shock. In the present study we investigated how Cl-amidine promotes survival, focusing on protective effects of Cl-amidine on immune response. We confirmed survival-improving effect of Cl-amidine and are the first to explore the role of Cl-amidine in immune response. CLP caused bone marrow (BM) and thymus atrophy, decreased innate immune cells in BM. CLP increased levels of cytokines (IL-1β, IL-6, and TNF-α) and bacteria load in blood/liver. In primary splenocyte culture, lipopolysaccharide increased TNF-α production. In contrast, Cl-amidine attenuated these CLP and lipopolysaccharide-induced alterations. Moreover, Cl-amidine increased circulating monocytes. Collectively, our results demonstrate Cl-amidine plays protective roles by significantly decreasing BM and thymus atrophy, restoring innate immune cells in BM, increasing blood monocytes and blood/liver bacteria clearance, and attenuating pro-inflammatory cytokine production in a murine model of lethal sepsis.

Sepsis causes more than 225,000 deaths per year in the United States^1^. With many failed clinical-trials, it is urgent to explore more effective therapeutic approaches in order to improve the prognosis of severe sepsis and septic shock^2^. Protein-arginine deiminase (PAD) is an enzyme that catalyzes posttranslational modification of arginine called deimination or citrullination. It converts arginine and water into citrulline and ammonia.

PAD was recently revealed to play a key role in the etiology of some types of cancer such as leukemia, breast cancer and colon adenocarcinoma, and many autoimmune diseases, e.g., rheumatoid arthritis^3^,^4^. Inhibitor of PAD was proved to exhibit enhanced cell killing in PAD-expressing osteosarcoma cells and block the formation of neutrophil extracellular traps^5^. PAD inhibitor YW3-56 activates 53 target genes, and effectively inhibits the growth of triple-negative breast xenograft tumors in nude mice. YW3-56-mediated cell death also featured mitochondria depletion and autophagy perturbation^6^. Chloramidine (Cl-amidine), an inhibitor of PAD, reduced the clinical signs and symptoms of colitis, and induced apoptosis of inflammatory cells *in vitro* and *in vivo*^7^. We previously demonstrated that inhibition of PAD by Cl-amidine markedly suppresses the production of citrullinated histone H3 (CitH3), and neutralization of CitH3 improves survival markedly in septic mice. However, the mechanisms underlying effects of Cl-amidine on severe sepsis and septic shock remain unknown.

Immune organs, such as bone marrow and thymus, are maturation centers for B and T lymphocytes in adaptive immune system. Bone marrow hematopoietic stem cells also give rise to blood cells. Immune cells in circulation, especially monocytes and neutrophils, are essential components of innate immune system to eliminate pathogens and harmful molecules. Bacteria are the most common pathogens invading host during sepsis. Evaluating bacteria load provides guidance to diagnose infection and determine the severity and prognosis of sepsis. In addition, excessive inflammatory responses cause cellular and organ damage during severe sepsis and septic shock.

Therefore, the current study aimed to investigate effects of Cl-amidine on survival outcomes, and explore effects of Cl-amidine on atrophy of immune organs, composition of immune cells in bone marrow and blood, bacteria clearance in liver and blood, and inflammatory responses.

## Results

### Cl-amidine protected mice from sepsis-induced lethality

**Figure 1:**
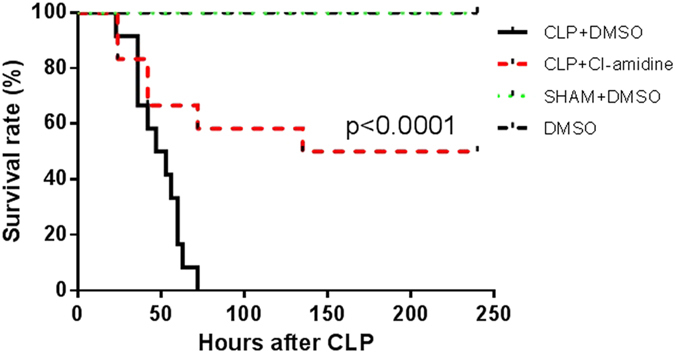
Cl-amidine protected mice from sepsis-induced lethality. Mice were intraperitoneally administered 40 mg/kg of Cl-amidine dissolved in DMSO or vehicle DMSO 1 h after CLP. DMSO was also injected into normal mice (DMSO group) and mice that were operated but not subjected to CLP (Sham + DMSO group). CLP-subjected animals treated with PAD inhibitor Cl-amidine had higher long-term survival rate compared to CLP + DMSO group (50% versus 0% survival). CLP: cecal ligation and puncture. DMSO: dimethyl sulfoxide.

Mice were intraperitoneally administered Cl-amidine (40 mg/kg) dissolved in DMSO or vehicle DMSO 1 h after CLP. Animals that were operated without CLP served as sham. DMSO was also injected into normal mice (DMSO group) and mice that were operated but not subjected to CLP (Sham + DMSO group). Animals in CLP + DMSO group deceased within 3 days, and all animals in DMSO and Sham+DMSO groups survived. CLP-subjected animals treated with PAD inhibitor Cl-amidine had higher long-term survival rate compared to CLP + DMSO group (50% vs. 0%, *p* < 0.0001; Figure 1).

### Cl-amidine decreased bone marrow atrophy and restored innate immune cells in bone marrow

**Figure 2:**
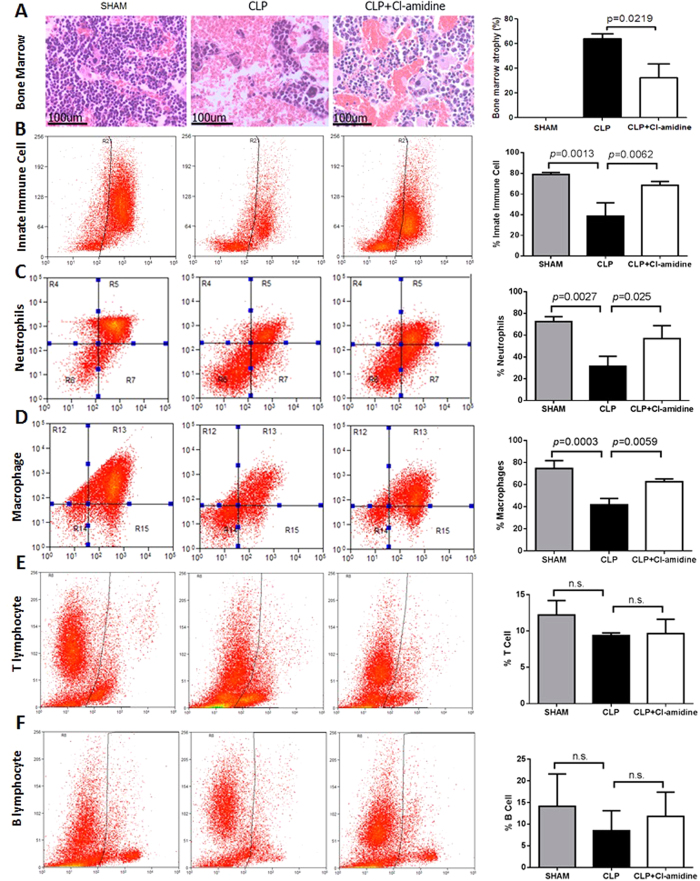
Cl-amidine decreased bone marrow atrophy and restored innate immune cells in bone marrow during severe sepsis. (**A**) Representative images from 3 groups (Sham, CLP + DMSO, and CLP + CL-amidine) are presented, and pathological scores for bone marrow atrophy were graded according to the diameter proportion of veins to bone marrow cells on a scale of 0% to 100% (n = 6/group). H&E staining: magnification ×40. **(B–F)** Long bones were harvested from sham animals and animals treated with or without Cl-amidine at 48 h after CLP (n = 3 animals per group). The bone marrow was prepared and then subjected to flow cytometry, after being stained with different antibodies: CD11b^+^ for innate immune cells **(B)**, CD11b^+^Gr-1^+^ for neutrophils **(C)**, CD11b^+^F4/80^+^ for macrophages **(D)**, CD3^+^ for T lymphocytes **(E)**, and B220^+^ for B cells **(F)**. Compared to CLP group, treatment with Cl-amidine significantly restored innate immune cells, including neutrophils and macrophages, in bone marrow. Data presented as mean ± SEM. CLP: cecal ligation and puncture. DMSO: dimethyl sulfoxide. H&E: Hematoxylin and eosin. n.s.: not statistically significant.

Tissue samples of long bones (femur and tibia) were harvested for histological analysis at 48 h after CLP. Hematoxylin and eosin (H&E) staining was utilized to analyze the degree of bone marrow atrophy, which was assessed on a scale of 0% to 100% according to the diameter proportion of veins to bone marrow cells. DMSO vehicle group had increased diameter proportion of veins to bone marrow cells, whereas Cl-amidine decreased the proportion (degree of bone marrow atrophy: 64.1 ± 3.9 vs. 32.5 ± 11.1, *p* = 0.0219; Figure 2). In addition, flow cytometry analysis showed that Cl-amidine treatment affected population of immune cells in bone marrow. Compared to Sham group, animals from vehicle-treated group showed decrease of percentage of innate immune cells (CD11b^+^, 78.9% ± 2.0% vs. 38.7% ± 12.9%, *p* = 0.0013) in bone marrow, while Cl-amidine restored the innate immune cells (68.5% ± 3.7% vs. 38.7% ± 12.9%, *p* = 0.0062, Fig. 2B). Both neutrophils and macrophages in bone marrow were substantially lost in animals from CLP groups in comparison to that in Sham group (CD11b^+^Gr-1^+^, 72.6% ± 4.5% vs. 31.6% ± 9.2%, *p* = 0.0027, and CD11b^+^F4/80^+^, 74.6% ± 7.1% vs. 41.8% ± 5.6%, *p* = 0.0006, respectively). Treatment with Cl-amidine significantly restored neutrophils (56.8% ± 12% vs. 31.6% ± 9.2%, *p* = 0.025, Fig. 2C) and macrophages (62.7% ± 2.5% vs. 41.8% ± 5.6%, *p* = 0.0059, Fig. 2D). No differences were found in the percentages of B (Fig. 2F) and T lymphocytes (Fig. 2E) among the three groups.

### Cl-amidine decreased thymic cortex atrophy during severe sepsis

**Figure 3:**
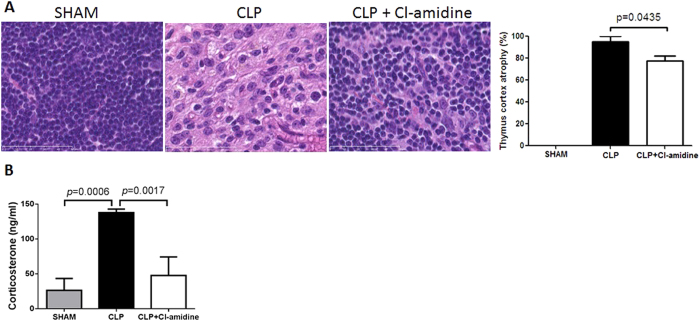
Cl-amidine decreased thymic cortex atrophy and blood levels of corticosterone during severe sepsis (H&E staining, magnification 100×). (**A**) Representative images from 3 groups of Sham, CLP + DMSO, and CLP + CL-amidine are presented, and pathological scores for thymic cortex atrophy were graded on a scale of 0% to 100% (n = 6/group). (**B**) Blood was collected from sham animals and animals treated with or without Cl-amidine at 48 h after CLP. Concentration of corticosterone was measured by ELISA (n = 3 animals per group). Data presented as mean ± SEM. CLP: cecal ligation and puncture. DMSO: dimethyl sulfoxide. H&E: Hematoxylin and eosin.

Thymus was harvested for histological analysis at 48 h after CLP. H&E staining was utilized to analyze the degree of thymic cortex atrophy, which was graded on a scale of 0% to 100% according to the degree of thymocyte depletion in the cortex. The degree of thymic atrophy was scored according to the color of thymic cortex after H&E staining. Thymic cortex was stained purple in sham group. In CLP group, the cortex appeared pink. These indicated that cells were depleted in thymic cortex of CLP-treated animals. However, compared to CLP group, thymic cortex was stained less pink, indicating that there was less thymic atrophy in CLP+Cl-amidine group (95.1 ± 5.1 vs. 77.5 ± 4.8, *p* = 0.0435; Fig. 3A).

A previously published study showed that thymic atrophy is associated with increased stress response during septic shock. In the current study, we measured levels of corticosterone in blood of mice treated with or without Cl-amidine after CLP. As shown in Fig. 3B, CLP induced dramatic increase of corticosterone compared to that in Sham group (137.9 ± 4.9 vs. 26.5 ± 17ng/ml, *p* = 0.0006). Treatment with Cl-amidine significantly attenuated CLP-induced elevated corticosterone (47.7 ± 26.6 vs. 137.9 ± 4.9 ng/ml, *p* = 0.0017).

### Effects of Cl-amidine on the number and percentage of blood cells

**Figure 4:**
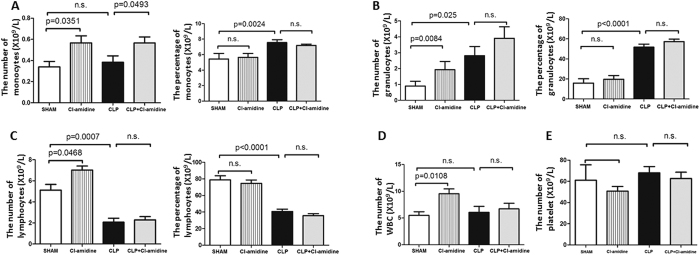
Cl-amidine altered the blood cell composition in sham-operated and CLP-subjected animals. (**A**) Cl-amidine increased the number of monocytes in sham-operated and CLP-subjected animals. (**B**) Effects of Cl-amidine on the number and percentages of granulocytes in circulation. (**C**) Effects of Cl-amidine on the number and percentages of lymphocytes in circulation. (**D**) Effects of Cl-amidine on the number of white blood cells in circulation. (**E**) Effects of Cl-amidine on the number of platelets in circulation. Peripheral blood samples were obtained at 48 h after CLP (means ± SEM, n = 8/group). n.s.: not statistically significant. CLP: cecal ligation and puncture.

Peripheral blood samples were obtained at 48 h after CLP, and analyzed by veterinary Hematrue hematology bench top analyzer. Cl-amidine increased the number of monocytes in sham-operated (0.6 ± 0.1 vs. 0.3 ± 0.2, *p* = 0.0351; Fig. 4A) and CLP-subjected animals (0.6 ± 0.1 vs. 0.4 ± 0.2, *p* = 0.0493; Fig. 4A). However, the percentages of monocytes in white blood cells were not altered by Cl-amidine. In addition, Cl-amidine increased the number of granulocytes significantly in sham-operated animals (3.9 ± 0.7 vs. 0.9 ± 0.4, *p* = 0.0084; Fig. 4B), but it did not increase the number significantly in CLP-subjected animals. Cl-amidine did not change the percentages of granulocytes in white blood cells. Similarly, Cl-amidine increased the number of lymphocytes markedly in sham-operated animals (7.0 ± 0.4 vs. 5.1 ± 0.6, *p* = 0.0468; Fig. 4C), but it did not increase the number significantly in CLP-subjected animals. Cl-amidine did not alter the percentages of lymphocytes in white blood cells. Moreover, Cl-amidine increased the number of white blood cells in sham-operated animals (9.5 ± 0.9 vs. 5.5 ± 0.8, *p* = 0.0108; Fig. 4D), but it did not increase the number markedly in CLP-subjected animals. Meanwhile, Cl-amidine did not alter the number or percentages of platelets (Fig. 4E).

### Cl-amidine increased bacteria clearance in liver and peripheral blood after CLP

**Figure 5:**
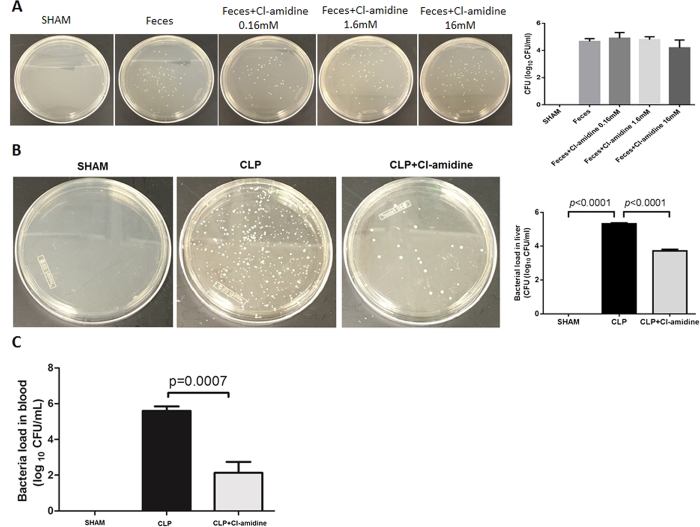
Cl-amidine increased bacteria clearance in liver and blood after CLP. (**A**) Cl-amidine itself has no antibiotic effect. Cecal feces of mice were suspended and diluted in normal saline with or without Cl-amidine. **(B)** Livers of animals from the 3 groups were harvested at 48 h after CLP. Supernatant was made after homogenation and centrifugation. Equal amount of supernatant was spread on agar plates for colony formation. In (**A**,**B**), representative colonies on agar plates were shown on the left panel. Statistical analysis of the colony formation was shown on the right panel (n = 3 animals per group). (**C**) Whole blood samples were obtained at 48 h after CLP. The number of bacterial colonies was assessed (n = 5/group). Data presented as mean ± SEM. CFU: colony forming units. CLP: cecal ligation and puncture.

First, we examined whether Cl-amidine could directly affect bacterial growth. Our data showed that Cl-amidine had no antimicrobial effect (Fig. 5A). To further assess whether Cl-amidine might increase bacterial clearance, we examined bacterial load in liver and peripheral blood at 48 h after CLP.

As shown in Fig. 5B, CLP increased bacterial load in liver compared to the Sham group (5.3 ± 0.1 vs. 0 ± 0 log_10_ CFU/ml, *p* < 0.0001), whereas treatment with Cl-amidine significantly enhanced bacterial clearance compared to that from CLP group (3.7 ± 0.2 vs. 5.3 ± 0.1 log_10_ CFU/ml, *p *< 0.0001). The similar results were also found from the bacterial clearance in blood (2.1 ± 0.6 vs. 5.6 ± 0.2 log_10_ CFU/mL, *p* = 0.0007; Fig. 5C).

### Cl-amidine attenuated CLP/LPS-induced pro-inflammatory cytokine production

**Figure 6:**
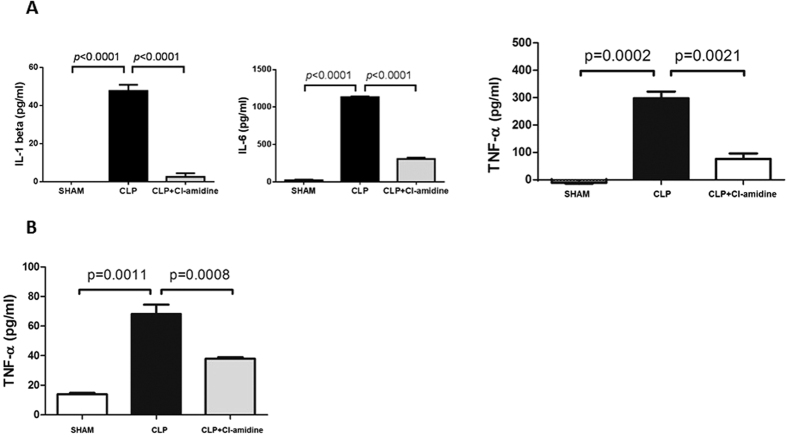
Cl-amidine attenuated CLP/LPS-induced pro-inflammatory cytokine production. (**A**) After CLP, blood samples were collected at 24 h (for TNF-α) or 48 h (for IL-1β and IL-6) for measurement of cytokine concentration (n = 3 animals per group). (**B**) Mouse primary splenocytes were treated with 1 μg/mL of LPS in the presence or absence of 10 μM of Cl-amidine (n = 5/group). The pro-inflammatory cytokines were measured by ELISA. Data presented as mean ± SEM. IL-1β: interleukin-1β. IL-6: interleukin-6. TNF-α: tumor necrosis factor-α. CLP: cecal ligation and puncture. LPS: lipopolysaccharide.

We assessed effect of Cl-amidine on pro-inflammatory cytokines (IL-1β, IL-6, and TNF-α) expression. Blood samples were collected at different time points after CLP. Mouse primary splenocytes were treated with 1 μg/mL LPS and 10 μM Cl-amidine. Cell culture supernatant was collected at 6 h after treatment. Plasma and cell culture supernatant were assayed for TNF-α by ELISA. At 24 h after CLP, the concentration of TNF-α in the plasma decreased in Cl-amidine group compared to DMSO vehicle group (76.2 ± 19.9 vs. 298.3 ± 24.6 pg/mL, *p* = 0.0021; Fig. 6A). In the cell culture, the concentration of TNF-α supernatant decreased in Cl-amidine group compared to LPS alone group at 6 h after LPS insult (38.0 ± 1.0 versus 68.1 ± 6.4 pg/mL, *p* = 0.0008; Fig. 6B). As for other pro-inflammatory cytokines, we measured levels of IL-1β and IL-6 in blood at 48 h after CLP. Similar to TNF-α, CLP increased levels of IL-1β and IL-6 in blood (47.6 ± 5.5 vs. 0 ± 0 pg/mL, *p* < 0.0001 for IL-1β, 1133.2 ± 17.2 vs. 16.5 ± 9.5 pg/mL, *p* < 0.0001 for IL-6, Fig. 6A), and treatment with Cl-amidine significantly decreased concentration of these cytokines (2.5 ± 3.3 vs. 47.6 ± 5.5 pg/mL, *p* < 0.0001 for IL-1β, 305.2 ± 33 vs. 1133.2 ± 17.2 pg/mL, *p* < 0.0001 for IL-6, Fig. 6A).

## Discussion

In this study, we have demonstrated that Cl-amidine, an inhibitor of PAD, protected mice against sepsis-induced lethality. Cl-amidine decreased the atrophy of bone marrow and thymic cortex in septic animals. Cl-amidine increased the number of monocytes in sham-operated and CLP-subjected animals, and increased the number of granulocytes, and white blood cells in sham-operated animals. Moreover, Cl-amidine increased bacteria clearance in liver and blood, and attenuated the concentration of IL-1β, IL-6, and TNF-α in blood, and decreased TNF-α in cell culture supernatant.

PAD catalyzes the post-translational hydrolysis of arginine residues to form citrulline. Disruption of normal PAD activity plays a role in the pathogenesis of various inflammatory diseases such as rheumatoid arthritis^8^, ulcerative colitis^9^, multiple sclerosis and psoriasis^10^, Alzheimer’s disease^11^, and in many cancers^12^,^13^,^14^. In current study, we revealed that Cl-amidine (40 mg/kg), an inhibitor of PAD, protected mice against sepsis-induced lethality, and the 10-day survival rate was 50%. This is similar to what we have revealed in mice treated with 80 mg/kg of Cl-amidine (10-day survival rate: 42.5%)^15^. Since PAD participates in a number of molecular processes, lower dose Cl-amidine may cause fewer side effects.

In current study we unveiled that Cl-amidine increased the number of granulocytes, and white blood cells in circulation. It is possible that inhibition of PAD regulates the differentiation of bone marrow hematopoietic stem cells and immunity, since bone marrow has been thought to be a hematopoietic and immune regulatory organ^16^. Therefore, we evaluated bone marrow of CLP-subjected animals in the presence or absence of Cl-amidine treatment in current study. We found that CLP increased bone marrow atrophy, whereas Cl-amidine attenuated the damage. Moreover, flow cytometry analysis further showed that CLP decreased innate immune cells in bone marrow, including neutrophils and macrophages (Fig. 2B,C,D). In contrast, inhibition of PAD with Cl-amidine increased these cells in the immune organ.

A question raised here is whether Cl-amidine could affect macrophage activity and bacterial burden in organs such as liver. Lining the walls of the liver sinusoids that form part of the mononuclear phagocyte system, Kupffer cells are specialized macrophages located in the liver. We prepared liver homogenate and examined effect of Cl-amidine on bacterial load in liver and function of Kupffer cells. Logically, after the homogenization of liver tissue and centrifugation, bacteria in hepatic sinusoids or interstitial spaces would get into the supernatant and grow on agar plates. Therefore, culture of the homogenized liver could reflect the bacteria burden in liver affected by Cl-amidine in the CLP model. Our data imply that the decreased bacterial load in liver might result from the uptake of bacteria by Kupffer cells. Similar to liver, we found that Cl-amidine also reduced bacterial burden in circulation (Fig. 5C). Given the fact that Cl-amidine restored innate immune cells in bone marrow (Fig. 2B,C,D) and decreased bacterial load in blood (Fig. 5C), we could conclude that Cl-amidine can restore innate immunity to enhance bacteria clearance in both liver and blood.

It has been known that sepsis can cause severe and sustained biological stress to the host, leading to organ damage, for instance, thymic atrophy in our study. It was reported that any stress or imbalance between neuroendocrine and immune responses favoring a pro-inflammatory state may trigger organ dysfunction and progression of infection to organ damage and failure^17^. We have recently demonstrated that increased thymic atrophy was related to increased stress response during severe sepsis and septic shock, due to increased concentration of corticosterone in CLP-subjected mice^18^. In the current study, we found that the elevated levels of corticosterone in blood coincided with thymic atrophy. Moreover, our study further revealed that Cl-amidine treatment can decrease blood concentration of corticosterone and reduce the degree of thymic atrophy during the lethal septic shock.

Excessive cytokine production is detrimental to cells, tissue, and organs during sepsis^19^, and the attenuation of cytokine storm protects against inflammatory damage^20^,^21^,^22^,^23^. We unveiled that Cl-amidine attenuated production of cytokines (IL-β, IL-6, and TNF-α) in the plasma of CLP-subjected animals and expression of cytokine (TNF-α) in cell culture supernatant of LPS-insulted splenocytes. Evidence is scarce for effects of PAD inhibitors on inflammatory responses. Cl-amidine was reported to induce apoptosis of inflammatory cells and reduce the clinical signs and symptoms of colitis^7^. Thus, the apoptosis of inflammatory cells after Cl-amidine treatment may be one reason for the decreased cytokine production as shown in current study. In addition, Toll-like receptor (TLR) 4 signaling pathway involves in cytokine production during sepsis^24^. It is possible that PAD may involve in post-translational modification of some proteins in TLR4 signaling pathway, and Cl-amidine may target some key steps. Further investigation is needed to determine these molecular mechanisms.

This study has some limitations to be acknowledged. Selected cytokine and pathways were investigated in current study for logistical reasons. It is likely that many more molecules and pathways are affected by Cl-amidine treatment. More underlying molecular mechanisms need to be explored.

In summary, we have demonstrated that inhibition of PAD with Cl-amidine improves survival outcomes, decreases the atrophy of bone marrow and thymus, increases innate immune cells in bone marrow, enhance the number of monocytes and bacteria clearance in liver and blood, and attenuates pro-inflammatory cytokine production in a lethal mouse CLP model. Inhibition of PAD may represent a promising therapeutic target for severe sepsis and septic shock.

## Methods

### Cells and Reagents

Mouse primary splenocytes were prepared by homogenizing harvested spleens through 70 μm-nylon meshes. Erythrocytes in the cell mixture were lysed by red blood cell Lysis Buffer (Sigma Aldrich Inc., St. Louis, MO, USA). Cells were treated with 1 μg/mL of LPS (Sigma Aldrich Inc., St. Louis, MO, USA) or 1 μg/mL of LPS and 10 μM of Cl-amidine (EMD Millipore, Billerica, MA, USA). Dubelcco’s modified Eagle’s medium (DMEM), fetal bovine serum, glutamine, penicillin, and streptomycin (Invitrogen Inc., Grand Island, NY, USA) were used for cell culture. We chose the concentration of 10 μM in order to compare the anti-inflammatory effect of Cl-amidine with HDAC inhibitors we tested in previous studies^25^. All drugs were added at the same beginning point. Quantikine Enzyme-Linked Immunosorbent Assay (ELISA) Kits (R&D Systems Inc., Minneapolis, MN, USA) were used for measurement of IL-1β, IL-6, and TNF-α.

### Cecal Ligation and Puncture-induced Septic Model

Male C57BL/6J mice (18–26 gram) were purchased from the Jackson Laboratory, and housed for 3 days before manipulations. The murine model of CLP^26^ modified by our laboratory was used to induce fecal peritonitis. The peritoneal cavity was cut open under inhaled isoflurane anesthesia. Cecum was eviscerated, ligated below the ileocecal valve using 5–0 suture, and punctured through and through (2 holes) with a 20 gauge needle. The cecum was then squeezed to expel a small amount of fecal material and returned to peritoneal cavity. The abdominal incision was sutured in two layers with 4-0 silk suture. Animals were resuscitated by injecting 1 mL saline subcutaneously. Sham-operated animals were handled in similar manner, but the cecum was neither ligated nor punctured. Surgeries were carried out under anesthesia, and efforts were made to minimize animal suffering. All experiments on mice were performed in accordance with the guidelines approved by the Animal Review Committee at Massachusetts General Hospital and University of Michigan.

### *In Vivo* Experimental Design

Experiment I: mice were injected with intra-peritoneal Cl-amidine dissolved in DMSO (40 mg/kg), or vehicle DMSO 1 h after CLP. DMSO was also injected into mice that were not subjected to CLP (n = 12/group). Mortality was monitored for 10 days after surgeries. Experiment II: animals were randomly assigned into three groups (n = 13/group): (a) Sham-operation group (SHAM); (b) DMSO vehicle treatment group (CLP + DMSO); and (c) Cl-amidine treatment group (CLP + Cl-amidine). All Cl-amidine treatment was given only once unless otherwise noted. Sham-operation group had laparotomy and intestinal manipulation, but no cecal ligation or puncture. Animals were sacrificed at 24 h (n = 5/group) and 48 h (n = 8/group) after CLP. Femur, tibia, and thymus were harvested at 48 h. They were then fixed in 10% buffered formalin for histological analysis. Blood samples were collected by cardiac puncture at 24 h and 48 h.

### Flow Cytometry of Bone Marrow Cells

Bone marrow of mouse femur and tibia was harvested 48 hours after CLP. Bone marrow single-cell suspension was prepared as described previously^27^. Bone marrow cells were then washed, blocked with anti-mouse CD16/32 (eBiosicence, San Diego, CA), and stained with anti-mouse B220 PE-Cy7, CD3 APC-eFluor 780, CD11b FITC, Gr-1 PerCP-Cy5.5, and F4/80 Antigen APC (eBiosicence) for 30 minutes on ice. The cells were washed before flow cytometry analysis. Data were collected and analyzed on a MoFlo Astrios Summit (Beckman Coulter, Brea, CA).

### Histological Analysis

Tissue samples of long bones (femur and tibia) and thymus were harvested for histological analysis at 48 h after CLP. Samples were fixed in 10% buffered formalin, embedded in paraffin, sliced into 5-μm sections and stained with H&E. Atrophy of bone marrow and thymic cortex were graded by a pathologist blinded to group allocation. The degree of bone marrow and thymic cortex atrophy was graded on a scale of 0% to 100%. Zero % means “no atrophy”, and 100% means “complete atrophy”. Bone marrow atrophy was assessed according to the diameter proportion of veins to bone marrow cells, and thymic cortex atrophy was evaluated by the degree of thymocyte depletion in the cortex. The degree of thymic atrophy was scored according to the color of thymic cortex and clearness of corticomedullary junction after H&E staining.

### Peripheral Blood Analysis

Peripheral blood was obtained via cardiac puncture at 48 h after surgeries using 1 mL heparinized syringes. Three hundred μl aliquots were analyzed within 10 minutes of blood collection utilizing veterinary Hematrue hematology bench top analyzer (Heska Corporation, Loveland, CO, USA). The number and percentage of monocytes, granulocytes and lymphocytes in white blood cells and platelet count were detected.

### Bacteria Load Determination

Livers from Sham, CLP, and CLP + Cl-amidine groups were harvested, and blood samples were obtained by cardiac puncture at 48 h after CLP under sterile conditions. Same amounts of the liver tissues were homogenized, incubated at 37 °C for 1 h, and centrifuged at 500 g for 5 min. The supernatant of the tissue and blood samples were then properly diluted with sterile normal saline, and plated on tryptic soy agars (BD Difco Inc., Franklin Lakes, NJ). The agar plates were incubated at 37 °C for 24 h, and the number of bacterial colonies was calculated as colony forming units (CFU) and data were log transformed for statistical analysis (n = 5/group).

In the same way, we determined whether Cl-amidine has an antibiotic effect. First, we assume that the Cl-amidine injected intraperitoneally is completely absorbed into circulation. Therefore, blood concentration of Cl-amidine is about 1.6 mM according to the calculation formula – (drug dosage × mouse body weight)/(estimated mouse blood volume × drug molecular weight). Based on our assumption, we took three doses of Cl-amidine (0.16 mM, 1.6 mM, and 16 mM) and tested possible effect of the PAD inhibitor on bacterial-killing. Cecal feces were obtained from mice and properly diluted with normal saline. Feces suspension in presence or absence of Cl-amidine (0.16, 1.6, and 16 mM) was plated on agar plate.

### Cell Culture of Primary Splenocytes

Single splenocyte suspension without red blood cells was prepared as described above. Mouse primary splenocytes were cultured in DMEM supplemented with fetal bovine serum (10%), penicillin (100 U/mL), streptomycin and glutamine (2 mM) at 37 °C in a cell incubator with 5% CO_2_. The cells were treated with 1 μg/mL of LPS or 1 μg/mL of LPS and 10 μM of Cl-amidine. Sham group had no drug added. The concentrations of TNF-α were detected in cell culture supernatant (n = 5/group).

### Cytokine Measurement

Concentrations of tumor necrosis factor-α (TNF-α) in the plasma and cell culture supernatant were detected utilizing Quantikine Enzyme-Linked Immunosorbent Assay (ELISA) Kit (R&D Systems Inc., Minneapolis, MN, USA) according to the kit instructions. Moreover, concentrations of interleukin-1 beta (IL-1beta) and interleukin-6 (IL-6) in the plasma were also measured utilizing Quantikine Enzyme-Linked Immunosorbent Assay (ELISA) Kit (R&D Systems Inc., Minneapolis, MN, USA) according to the kit instructions.

### Statistical Analysis

Results were demonstrated as mean ± SEM. Differences among 3 or more groups were assessed by one way analysis of variance (ANOVA) followed by Bonferroni post hoc testing for multiple comparisons. Student’s t-test was used to compare the differences between two groups. Kaplan-Meier method was used for survival rate, and differences were analyzed by log-rank test. Analyses were performed utilizing GraphPad Prism (GraphPad Software Inc., La Jolla, CA, USA). *P* values of 0.05 or less were considered significant.

## Additional Information

**How to cite this article**: Zhao, T. *et al*. Protective effect of Cl-amidine against CLP-induced lethal septic shock in mice. *Sci. Rep.* **6**, 36696; doi: 10.1038/srep36696 (2016).

**Publisher’s note**: Springer Nature remains neutral with regard to jurisdictional claims in published maps and institutional affiliations.
